# Impaired signaling pathways on Berardinelli–Seip congenital lipodystrophy macrophages during *Leishmania infantum* infection

**DOI:** 10.1038/s41598-024-61663-6

**Published:** 2024-05-16

**Authors:** Viviane Brito Nogueira, Carolina de Oliveira Mendes-Aguiar, Diego Gomes Teixeira, Francisco Paulo Freire-Neto, Leo Zenon Tassi, Leonardo Capistrano Ferreira, Mary Edythe Wilson, Josivan Gomes Lima, Selma Maria Bezerra Jeronimo

**Affiliations:** 1 Health Sciences Center, Federal University of Rio Grande do Norte, Natal, Brazil; 2 Institute of Tropical Medicine of Rio Grande do Norte, 655 Passeio dos Girassois, Natal, RN 59078190 Brazil; 3 Department of Biochemistry, Federal University of Rio Grande do Norte, Natal, Brazil; 4 Departments of Internal Medicine and Microbiology & Immunology, University of Iowa and the Veterans’ Affairs Medical Center, Iowa City, IA 52242 USA; 5 Department of Clinical Medicine, Onofre Lopes University Hospital, 620 Nilo Pecanha, Natal, RN 59013300 Brazil

**Keywords:** Gene expression, Infection, Metabolic disorders

## Abstract

Berardinelli–Seip congenital lipodystrophy (CGL), a rare autosomal recessive disorder, is characterized by a lack of adipose tissue. Infections are one of the major causes of CGL individuals’ premature death. The mechanisms that predispose to infections are poorly understood. We used *Leishmania infantum* as an in vitro model of intracellular infection to explore mechanisms underlying the CGL infection processes, and to understand the impact of host mutations on *Leishmania* survival, since this pathogen enters macrophages through specialized membrane lipid domains. The transcriptomic profiles of both uninfected and infected monocyte-derived macrophages (MDMs) from CGL (types 1 and 2) and controls were studied. MDMs infected with *L. infantum* showed significantly downregulated expression of genes associated with infection-response pathways (MHC-I, TCR-CD3, and granzymes). There was a transcriptomic signature in CGL cells associated with impaired membrane trafficking and signaling in response to infection, with concomitant changes in the expression of membrane-associated genes in parasites (e.g. δ-amastins). We identified pathways suggesting the lipid storage dysfunction led to changes in phospholipids expression and impaired responses to infection, including immune synapse (antigen presentation, IFN-γ signaling, JAK/STAT); endocytosis; NF-kappaB signaling; and phosphatidylinositol biosynthesis. In summary, lipid metabolism of the host plays an important role in determining antigen presentation pathways.

## Introduction

Berardinelli–Seip syndrome, a congenital generalized lipodystrophy (CGL), is an extremely rare autosomal recessive disease (3 cases/million), with only ~ 500 cases reported in the literature^1^. The disease is most commonly identified in Brazil, Lebanon, and Scandinavia^2,3^. In the state of Rio Grande do Norte, northeast Brazil, there are 32.3 CGL cases/million, accounting for one of the highest prevalence regions worldwide^4^. There are four well-described types of CGL; amongst these, types 1 and 2 are documented in the state of Rio Grande do Norte with over 60 documented cases^5^.

CGL Type 1 has causal mutations in the gene *AGPAT* that encodes a key enzyme in triglyceride biosynthesis (1-acyl-glycerol-3-phosphate-*O*-acyltransferase-2). *AGPAT* has 11 known isoforms, each encoded by a different gene. The AGPAT2 isoform is highly expressed in adipocytes, and its enzymatic activity is involved in adipocyte differentiation and regulating the early stages of adipogenesis^3,6,7^. Other isoforms are also expressed in adipose tissue, but at lower levels than AGPAT2. This mutation leads to impaired triglyceride and phospholipid biosynthesis in adipocytes.

Subjects in Rio Grande do Norte, Brazil, with Type 1 CGL harbor a mutation on chromosome 9q34, in AGPAT exon 5 (A748T—Lys216stop, rs138994150), whereas CGL Type 2 has a causal mutation in the *BSCL2* (located on chromosome 11q13, Exon 4, -insA > 543: 544, frameshift, -rs786205071). *BSCL2* encodes Seipin, a protein with a central role in fat storage in lipid droplets and in adipocyte maturation. Seipin is widely expressed in the central nervous system, where it plays a crucial role in both intellectual development and motor neuron function. Mutations in the seipin gene are also responsible for a group of neuronal motor neuropathies—called seipinopathies^8,9^.

Individuals with types 1 and 2 CGL usually present with nephropathy, liver disease, *acanthosis nigricans* bone cysts and heart disease, such as cardiomyopathy, and lipoatrophic diabetes (DM2)^2^. Those complications tend to occur at an early age, younger than 15 years. They also may have premature death, caused by liver disease or infections. Respiratory infections are the most common currently reported^10^. The mechanisms that predispose to infections are still poorly understood.

Leishmaniasis is a complex of diseases caused by species of the genus *Leishmania*. Some long-standing observations and some newer reports suggest that lipids and lipid metabolism may play an important role in the outcome of this infectious disease^11,12^. One of the clinical forms is visceral leishmaniasis (VL), a systemic and fatal disease in about 5% of the cases. VL is caused by *Leishmania infantum* in Brazil and in Europe^13^. The annual incidence of symptomatic human VL worldwide by all visceralizing species of *Leishmania* is estimated to be between 50,000 and 90,000, although this is likely an underestimate^14^. Many more individuals are infected without disease manifestations and may harbor the parasite for long. The *Leishmania* spp. are obligate intracellular parasites in their mammalian hosts, usually residing in tissue macrophages (Mø)^15^.

*Leishmania* spp. parasites establish their infection in phagocytes within phagocytophorous vacuoles induced through receptor-mediated phagocytosis, becoming encased in a vacuole surrounded by a host cell membrane. Virulent *Leishmania* spp. become coated with the complement component iC3b and are taken up through macrophage surface receptor CR3 (complement receptor 3) in concert with other receptors^16^. CR3 and other receptors are localized in host membrane caveolae, which are cholesterol-enriched membrane microdomains^17^. Parasites require caveolin-mediated phagocytosis to gain access to an intracellular host cell compartment in which they will survive^18^. *Leishmania* amastigotes appear to incorporate host glycosphingolipids into their membrane^19^. In addition, individuals with symptomatic VL present altered lipid profiles and metabolism^20^, with increase in triglycerides and decrease in cholesterol and HDL.

Although there are studies of knockout macrophages from murine models, mice respond differently from humans to infection with different *Leishmania* species, and often do not develop the metabolic impairment observed in other mammals in response to intracellular infection^21^. The specific metabolic defects present in CGL provide an opportunity to study the effects of lipids in leishmaniasis, using a natural human gene knockout that alters overall lipid storage and biosynthesis and potentially gain insight in understanding the susceptibility of CGL individuals to infections.

*L. infantum* was used here as an in vitro model of intracellular pathogen infection to explore mechanisms of infection in the face of aberrant host cell triglyceride synthesis or storage. The *Leishmania* spp. parasites depend on the host cell as the source of lipids during infection. Furthermore, during VL there is characteristic dysregulation of serum lipid profiles of the affected individual. The current study enabled us to investigate the effect of lipids on gene expression of the parasite in this context of altered triglyceride synthesis and lipid storage by the host and the host response to infection. We hypothesized that CGL cells might exhibit impaired antimicrobial responses due to the fact that phagocytosis, and the downstream microbicidal responses triggered by phagocytosis, depend upon host cell membrane lipids. We used monocyte-derived macrophages (MDMs) isolated from individuals with CGL, and exposed these to *L. infantum* promastigotes under conditions that induce phagocytosis. The results showed significant differential expression of genes (DEGs) associated with cell entry, immune synapse, signaling and associated functions in infected CGL host cells when compared to infected controls. DEGs from CGL type 1 and CGL type 2 are discussed separately and aggregated. Parallel changes in the expression of membrane-associated genes were also observed in phagocytosed parasites.

## Results

### Subject characteristics

Participants were individuals living in the state of Rio Grande do Norte of Brazil and were separated by groups: AGPAT2mut (n = 3, two females and one male; individuals diagnosed with CGL type 1, AGPAT2 mutation) and BSCL2mut (n = 3, two females and one male; individuals diagnosed with CGL type 2, *BSCL2* mutation), and WT (n = 4, two females and two males, Wild Type for CGL). The median ages were 16 (range 8 months to 22 years old) in CGL individuals, and 25 (range 18 to 30 years old) in controls. None of the participants had a history of visceral leishmaniasis. All resided in the state of Rio Grande do Norte which is highly endemic for VL, although most were not subject to high exposure situations. At the time of the study, with the exception of one control that had detectable anti-*Leishmania* antibodies, none of the other participants had a positive anti-*Leishmania* serological or positive *L. infantum* DNA test (see Supplementary Table S1). Blood monocyte-derived macrophages (MDMs) were incubated with stationary phase *L. infantum* promastigotes for 2 h and left for 72 h for assessing infection and *Leishmania* growth. We observed that the number of intracellular *Leishmania* did not differ statistically among the three groups of infected MDMs (see Supplementary Fig. S2).

### Transcriptomes of human MDMs with or without *L. infantum* infection

**Figure 1:**
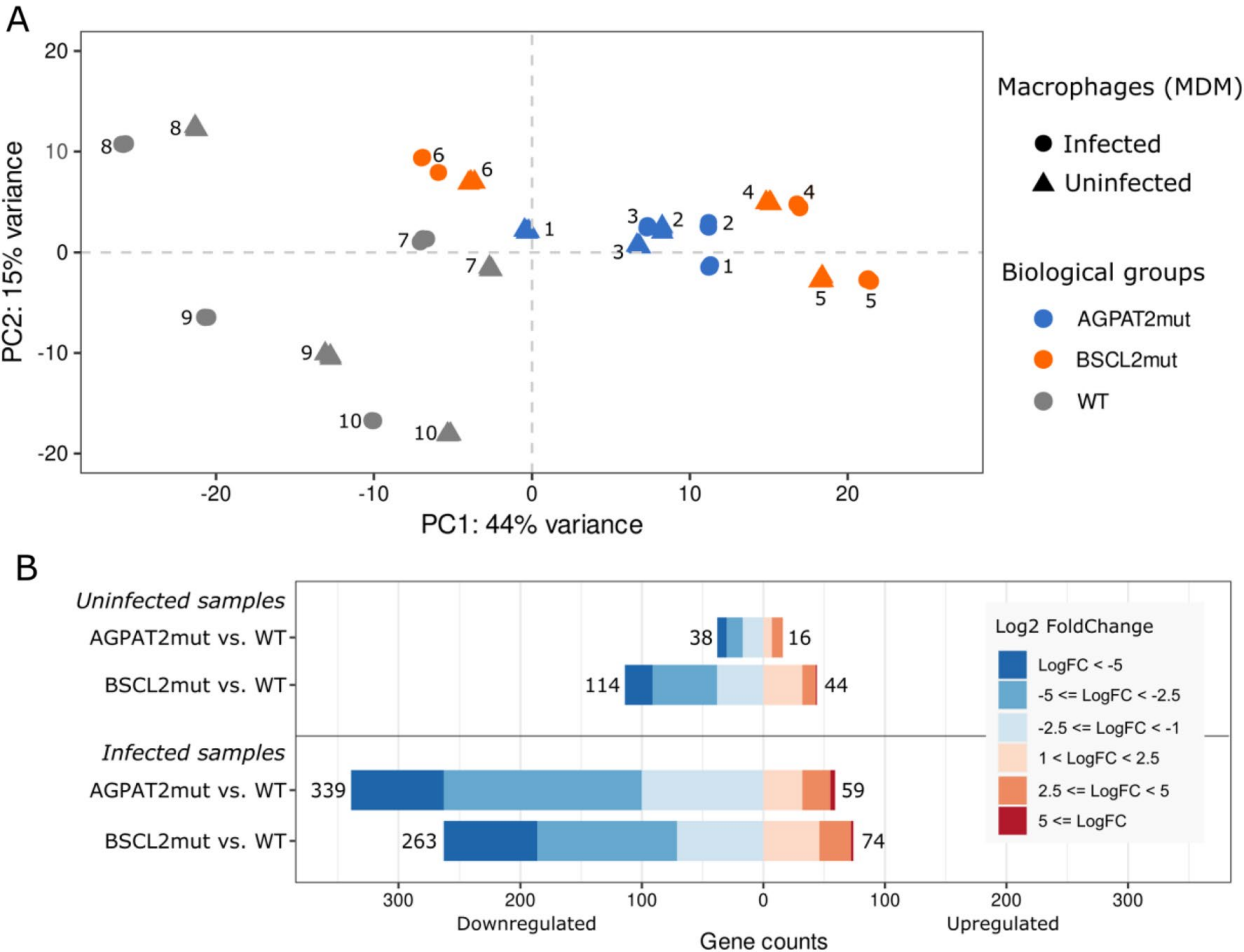
PCA over normalized gene expression values and DEGs. (**A**) PCA was calculated using the DESeq2 package, showing the spatial concentration of samples within the biological groups. For each sequenced biological sample, there are three experimental replicates represented in the PCA. (**B**) DEGs from RNA-seq in the four comparisons (Uninfected BSCL2mut vs. Uninfected WT, Uninfected AGPAT2mut vs. Uninfected WT, Infected BSCL2mut vs. Infected WT, and Infected AGPAT2mut vs. Infected WT). The color scheme represents the Log2 Fold Change of DEGs meeting the p-adjust threshold of < 0.05.

The genetically distinct groups were compared to discover global differences in gene expression induced by *L. infantum* infection. Patterns of DEGs in MDM of homozygotes with either the *BSCL2* or *AGPAT2* mutations clustered together and separately from WT control samples, regardless of their infection status. Principal component analysis (PCA) of these samples showed a separation between the biological groups (PC1) but not infection-associated responses (Fig. 1A).

Varied numbers of DEGs were found in the comparisons and the Fig. 1B depicts the number of DEGs present in the uninfected CGL cells compared to WT (AGPAT2mut had 54 DEGs, 16 upregulated; and BSCL2mut had 158 DEGs, 44 upregulated). Similarly, *L. infantum* infected CGL cells had a high number of DEGs (AGPAT2mut had 398 DEGs, 59 upregulated; and BSCL2mut had 337 DEGs, 74 upregulated). More detailed information can be found as Supplementary Table S2, in which a list of all DEGs for all comparisons is provided.

In the uninfected BSCL2mut some upregulated genes (*FADS2, EBP, FASN, DHCR7* and *TM7SF2*) were related to cholesterol metabolism, with functions in cholesterol synthesis, fatty acid metabolism, and lipid homeostasis. Clinically, disruptions result in the metabolic disorders, including hypercholesterolemia, which is seen in subjects with CGL.

#### AGPAT2 and BSCL2 mutations influencing the host response to *L. infantum* infection

**Figure 2:**
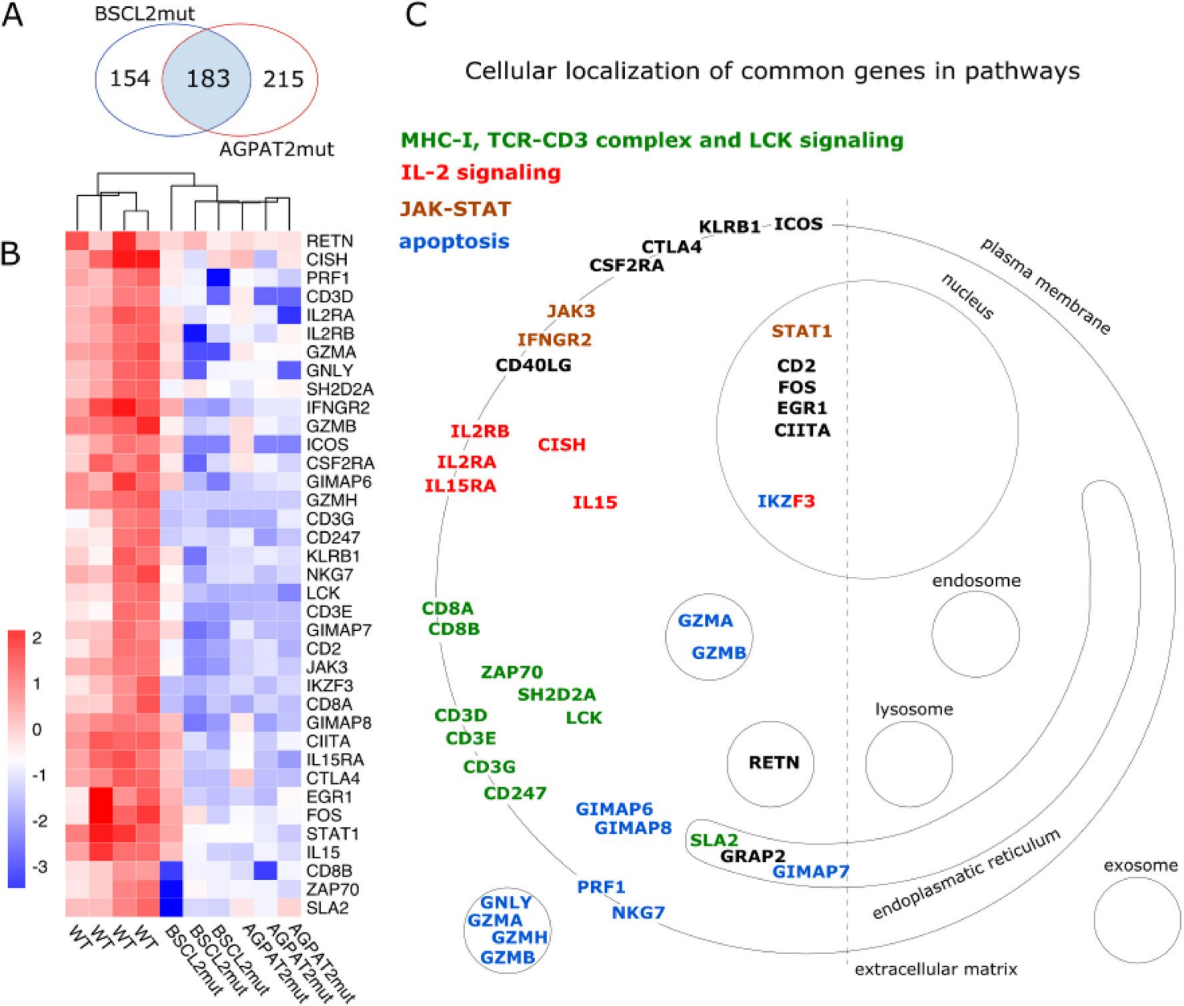
DEGs from AGPAT2mut and BSCL2mut when compared to WT in response to *L. infantum* infection. (**A**) Venn diagram of infected macrophages at two different comparisons. (**B**) Heatmap of common DEGs from AGPAT2mut and BSCL2mut vs. WT comparisons, selected by authors. All genes presented were downregulated in the both CGL types when compared to WT, and (**C**) Putative cellular localization of the same genes in (**B**).

**Figure 3:**
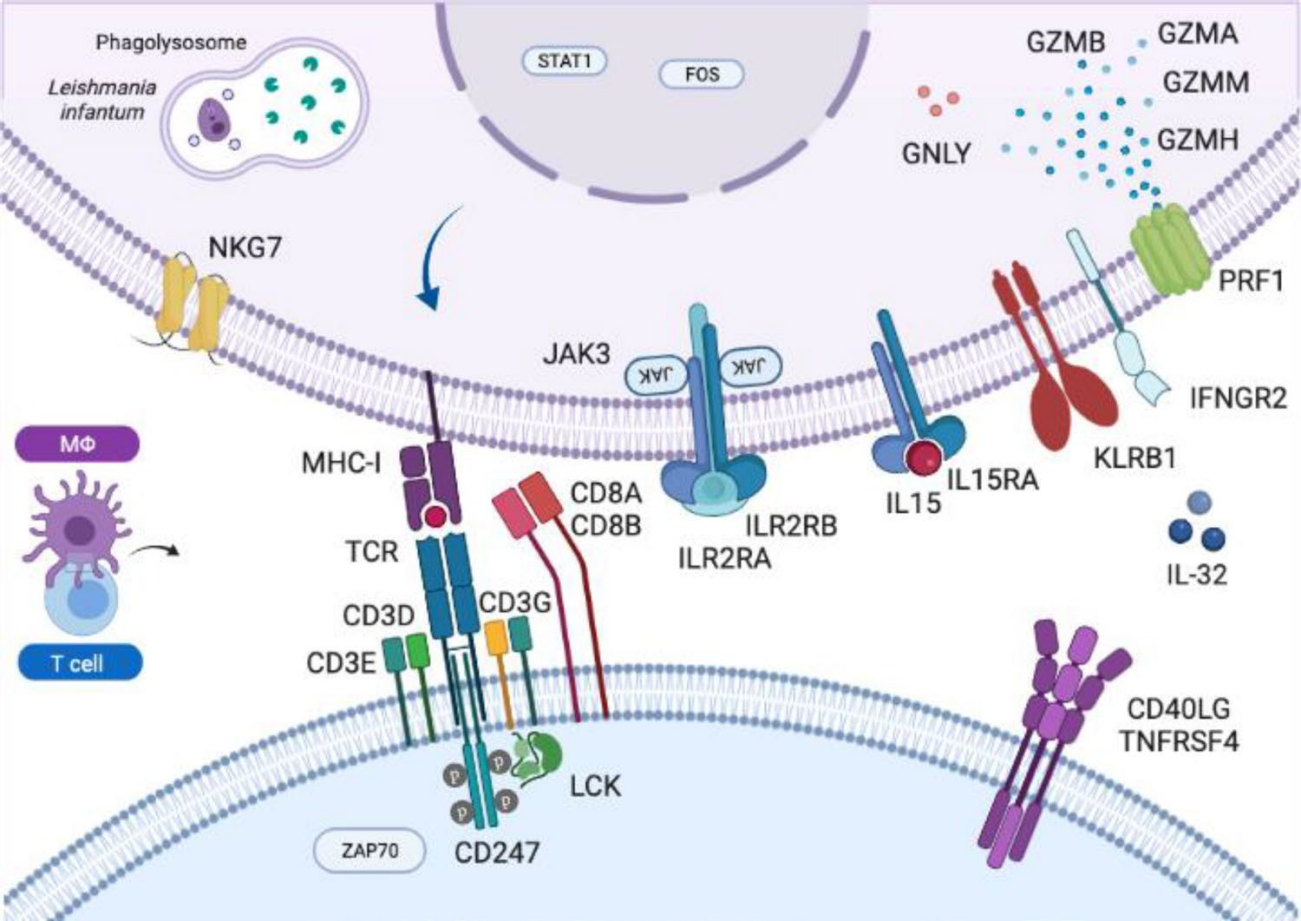
Putative roles of genes found in the gene expression analysis. Genes encoding all putative proteins represented here were significantly downregulated in both AGPAT2mut and BSCL2mut groups when compared to WT. The genes are possibly related to MHC-I, TCR-CD3 complex, LCK and IL-2 signaling, JAK/STAT, and apoptosis. More detailed information about differential gene expression can be found as Supplementary Table S2.

Common DEGs in the infected cells isolated from individuals with lipodystrophy and WT were compared to each other. There were 183 DEGs in common. Gene set enrichment analysis was performed (Fig. 2A,B). The putative cellular localization of DEGs is shown in Figs. 2C and 3.

MHC-I and TCR-CD3 complex genes were downregulated in infected MDMs from CGL individuals, both Types 1 and 2. The LCK, Janus kinase/signal transducer and activator of transcription (JAK-STAT), and IL-2 signaling pathways were also downregulated. Myeloid cytotoxic pathways (such as granzyme, perforin, and granulysin) were among the downregulated infection-response genes. The signaling pathways for T cell activation and apoptosis were also drastically downregulated in MDMs from individuals with CGL. In fact, there was an impairment, with both up and downregulated genes in the response to *L. infantum* infection in MDMs from individuals with lipodystrophy.

**Figure 4:**
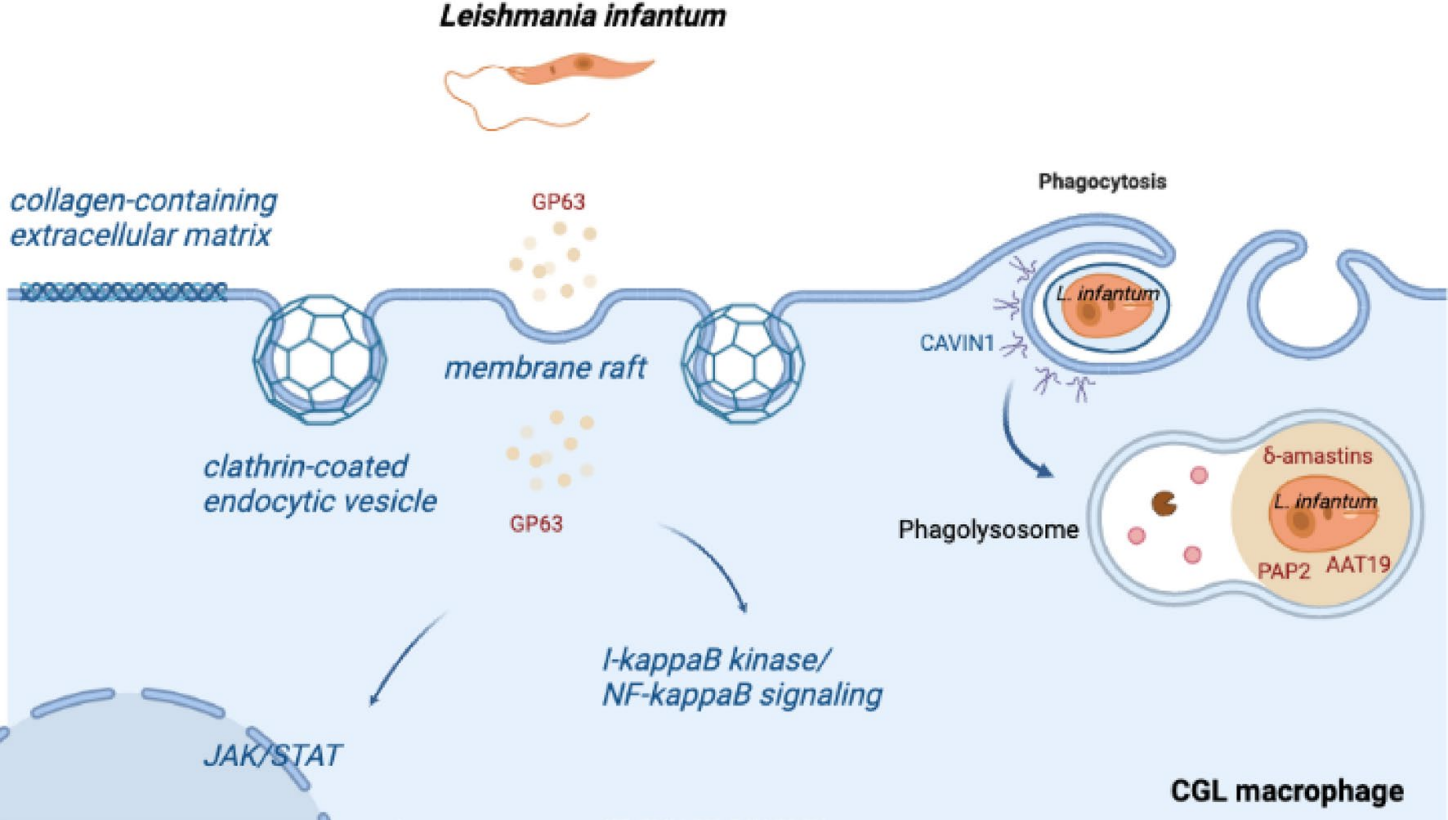
Schematic representation of host cells enriched pathways in response to infection and differential gene expression of *L. infantum.* All putative proteins or pathways represented here were found as significantly enriched in the Gene Ontology analysis or as a differentially expressed gene. More detailed information about the host enrichment analysis can be found as Supplementary Table S1. CAVIN1 is downregulated in AGPAT2mut host cells. For *L. infantum* expression, amastins were downregulated when infecting both AGPAT2mut and BSCL2mut groups; PAP2 and AAT19 are upregulated in BSCL2mut, and GP63 is upregulated in AGPAT2mut. More detailed information about the *L. infantum* gene expression is present in Table 1.

After assessing the common DEGs in the infected cells isolated from individuals with both CGL types and WT, the DEGs from Types 1 and 2 CGL were evaluated separately. There were commonly enriched categories (see Supplementary Table S3, tabs of common categories for Cellular Component, Molecular Function and Biological Process), e.g. *endocytic vesicle membrane* (GO:0030669, AGPAT2mut q-value = 0.0092, BSCL2mut q-value = 0.0059), *clathrin-coated endocytic vesicle* (GO:0045334, 7 genes in AGPAT2mut q-value = 0.0329, and 8 genes in BSCL2mut q-value = 0.0026), *antigen receptor-mediated signaling pathway* (GO:0050851, 29 genes in AGPAT2mut, q-value = 2.01e^−13^, and 33 genes in BSCL2mut, q-value = 11.06e^−18^), *antigen processing and presentation of peptide antigen *via* MHC class II* (GO:0002495, 4 genes in both AGPAT2mut qvalue = 0.244, and BSCL2mut qvalue = 0.0183), *toll-like receptor signaling pathway* (GO:0002224, 8 genes in AGPAT2mut, qvalue = 0.0202), and *regulation of phosphatidylinositol 3-kinase signaling* (GO:0014066: 8 genes in both AGPAT2mut qvalue = 0.0126 and BSCL2mut qvalue = 0.0053). Those categories are strongly related to membrane trafficking and signaling (Fig. 4). There were also commonly regulated categories relevant to immune response, including *immunological synapse* (GO:0001772: AGPAT2mut q-value = 7.22e^−06^, BSCL2mut q-value = 9.18e^−08^), and *immunoglobulin complex, circulating* (GO:0042571: AGPAT2mut q-value = 7.94e^−05^, BSCL2mut q-value = 1.65e^−09^). Other categories included *regulation of neuron death* (GO:1901214, 13 genes for both AGPAT2mut q-value = 0.0463, and BSCL2mut q-value = 0.0199) represented in the enrichment analyses for Type 1 and 2 separately.

Exclusively in the Type 1 lipodystrophy (AGPAT2mut) comparison, there was a *collagen-containing extracellular matrix* (GO:0062023, q-value = 0.0269) category with *COL4A1* and *FNDC5* genes upregulated, and *COL1A1*, *WNT10A* and *WNT5A* genes downregulated. In Type 2 lipodystrophy (BSCL2mut), there was the *membrane raft* (GO:0045121, q-value = 0.0026) category enriched, which means this category was present only in Type 2 comparison. In addition, lipodystrophy-related genes were modulated: *CAVIN1* (downregulated in infected AGPAT2mut vs. WT) and *FOS* (downregulated in AGPAT2mut and BSCL2mut vs. WT). More detailed information can be found as Supplementary Table S3, in which a list of all enrichment analyses (cellular component, molecular process, and biological process from Gene Ontology) for all infected comparisons is provided.

In the infected AGPAT2mut, the category *inositol lipid-mediated signaling* (GO:0048017, qvalue = 0.0062) was enriched with the downregulated genes *HTR2A* and *ERBB4*, and the upregulated genes *PTAFR*, *GATA3*, *ERBB3*, *NLRC3*, *CCL5*, *PPP1R16B*, *KIT*, *NCF1* and *SEMA4D*. Categories of *lipopolysaccharide-mediated signaling pathway* (GO:0031663, 0.0337) and *regulation of phospholipase activity* (GO:0010517, qvalue = 0.0485) were also potentially related to lipid metabolism. In the infected BSCL2mut, the categories *phosphatidylinositol-mediated signaling* (GO:0048015, qvalue = 0.0073) and *inositol lipid-mediated signaling* (the same as in AGPAT2mut comparison, qvalue = 0.0084).

A comparison between CGL Type 1 and Type 2 can illuminate the pathogenesis of lipodystrophy in general. Relevant to this, there were 18 genes that were differentially expressed in the infected AGPAT2mut vs. infected BSCL2mut, 9 genes downregulated and 9 genes upregulated, and are involved in a range of cellular processes, e.g. signal transduction, metabolism, immune response, and development (see Supplementary Table S2).

#### Uninfected macrophages: background gene expression

When MDMs from AGPAT2mut individuals were compared to controls, there were 54 DEGs, 38 of which were downregulated. Of those 54 DEGs, *PTPRF* was upregulated, whereas *SERPINA1*, *IGFBP4*, and *CTLA4* were downregulated. These genes are related to type 2 diabetes mellitus (DM2), which is widely present in individuals with lipodystrophy. *FZD2* and *HLA-F* (both downregulated) and *TFRC* (upregulated) are related to clathrin-coated endocytic membrane vesicles and endocytosis and were differentially expressed. *EGR1* and *FOS* (both downregulated) are related to leptin metabolism.

The comparison between uninfected BSCL2mut vs. WT revealed 158 DEGs, 114 of which were downregulated. Many significantly regulated categories in the enrichment analysis of uninfected samples involved immune responses (see Supplementary Table S4). Two interesting categories are *neuron death* (GO:0070997, q-value = 0.022) which contains 9 genes, 5 of which were downregulated, and *phagocytosis* (GO:0006909, q-value = 2.868e−05) that contains 13 genes, 12 of which were downregulated. Genes involved in the cognitive impairment *MAG, GDF15, GPR176*, and *EPHB1* (upregulated), *BCL11B, GIMAP7,* and *ACOD1* (downregulated) were differentially expressed. Besides, the categories *cholesterol biosynthetic process* (GO:0006695, q-value = 0.0161) and *response to lipopolysaccharide* (GO:0032496, qvalue = 0.0176) were enriched, potentially related to lipid metabolism.

Additionally, there were 12 DEGs in the uninfected AGPAT2mut vs. uninfected BSCL2mut: *LYVE1, FLG, ADAM22, SERPINB7, C6orf47, VLDLR, TAPT1, ZFAT, IL17F, TPSAB1, WDR72* and *TPSB2*. Some of those genes are related to endocytosis, and trafficking through the Golgi.

### *L. infantum* differential gene expression and metatranscriptomics

**Figure 5:**
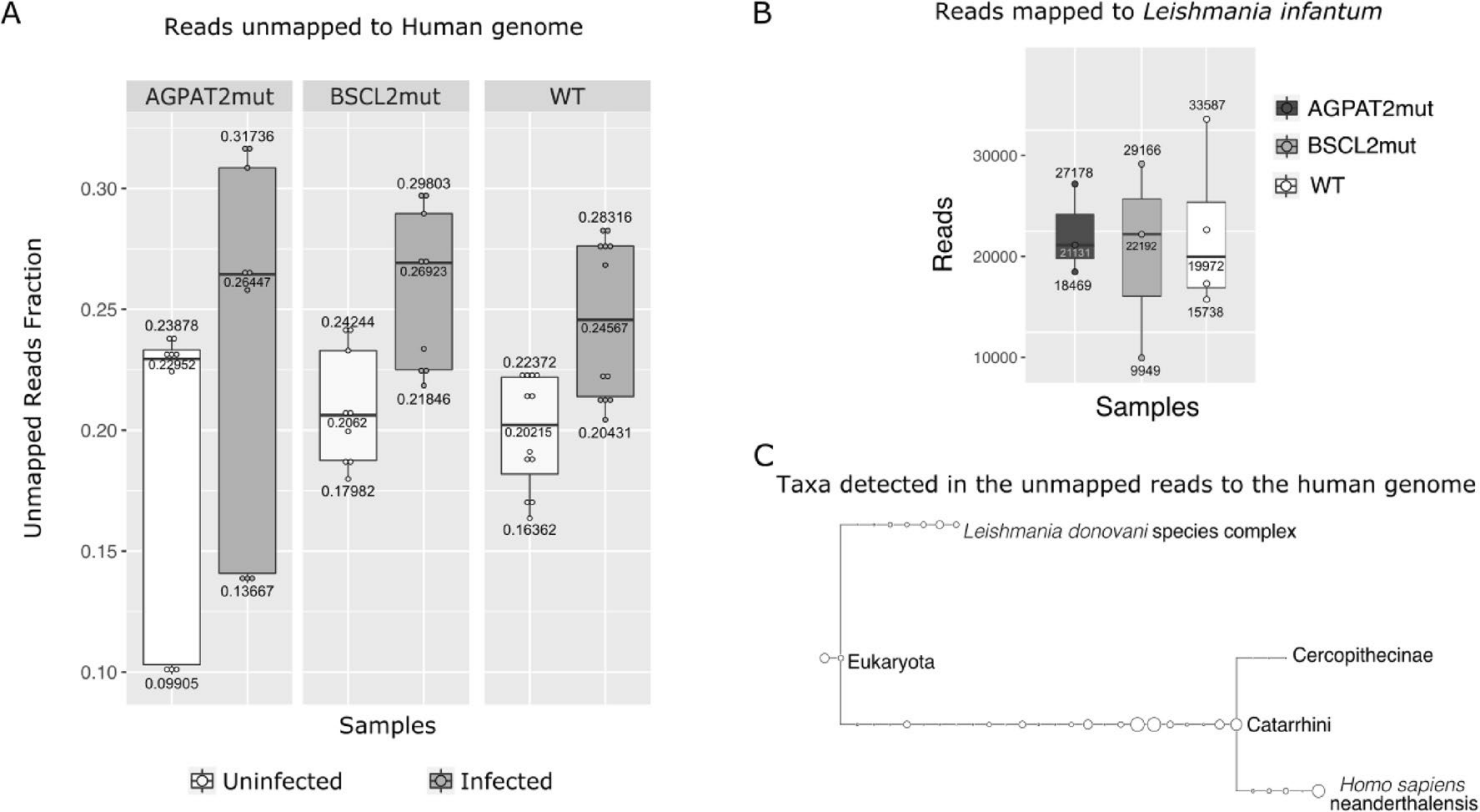
Metatranscriptomics of biological groups. Infection experiments were controlled for a parasite-host cell ratio of 5:1. The quantitative parasite load was not quantified. (**A**) Reads that could not be aligned to the Human genome, for all groups. (**B**) Reads that could be aligned with the *Leishmania infantum* genome. (**C**) Overview of the taxonomy identified for reads not aligned to the human genome. Taxonomy was obtained via BLASTx in Megan6, using the LCA (Lowest common ancestor) algorithm. The graphical representation was made using the Compute core biome function, the threshold of 50%. The proportion of the nodes is according to the default display mode (sqrt).

A mean of 22.45% ± 4.53 of reads were unmapped to the human genome in the samples processed in this study. Unmapped genes in samples infected with *L. infantum* were 24.61% ± 3.91 and unmapped genes in uninfected samples were 20.04% ± 3.93 of the reads. We performed metatranscriptomics on the unmapped genes (Fig. 5A). The unmapped reads aligned to the *Leishmania* complex (Fig. 5B) or to several primates (Fig. 5C). The reads that mapped to the *L. infantum* genome were subjected to differential expression analysis (see Supplementary Fig. S3).

In the comparison Infected AGPAT2mut vs. WT, 4 genes were upregulated and 8 were downregulated (Table 1). We found upregulation of the virulence factor Leishmanolysin (*GP63*) and protein kinase, and downregulation of Lipase (class 3), protein kinase and Amastin surface glycoprotein, and amastin-like surface protein in AGPAT2mut.

**Table 1:** *L. infantum* differentially expressed genes, when infecting AGPAT2mut vs. WT or BSCL2mut vs. WT MDMs.

Genotype	GeneID	Lmj homolog	Locustag	Description	padj	Log2FC
AGPAT2mut	LINF_040006100	LmjF.04.0100	LINJ_04_0110	Hypothetical protein—conserved	0.0128	1.0511
AGPAT2mut	LINF_100010400	LmjF.10.0480	LINJ_10_0502	GP63—leishmanolysin	0.0058	1.1600
AGPAT2mut	LINF_220017500	LmjF.22.1150	LINJ_22_0970	Protein kinase domain containing protein	0.0335	1.0077
AGPAT2mut	LINF_260031500	LmjF.26.2570	LINJ_26_2600	Protein kinase^P^	0.0035	1.2458
AGPAT2mut	LINF_130006900	LmjF.13.0200	LINJ_13_0200	Lipase (class 3)^P^	0.0035	− 1.1560
AGPAT2mut	LINF_150012100	NA	NA	NA	0.0112	− 1.0770
AGPAT2mut	LINF_170010200	LmjF.17.0390	LINJ_17_0440	Protein kinase^P^	0.0126	− 1.0570
AGPAT2mut	LINF_310022400	LmjF.31.1510	LINJ_31_1540	Hypothetical protein—conserved	0.0129	− 1.0158
AGPAT2mut	LINF_340017500	LmjF.34.1080	LINJ_34_1150	Amastin-like surface protein^P^	0.0065	− 1.1021
AGPAT2mut	LINF_340023000	NA	LINJ_34_1680	Amastin surface glycoprotein^P^	0.0126	− 1.0993
AGPAT2mut	LINF_340034800	NA	LINJ_34_4345	Amastin surface glycoprotein^P^	0.0035	− 1.2827
AGPAT2mut	LINF_340034900	NA	LINJ_34_4350	Amastin surface glycoprotein^P^	0.0012	− 1.2907
BSCL2mut	LINF_340024500	LmjF.34.1560^a^	LinJ_34_1705	Amastin-like surface protein^P^	0.0073	− 1.2282
BSCL2mut	LINF_340015600	NA	LinJ_34_1016	Amastin surface glycoprotein^P^	0.0026	− 1.0843
BSCL2mut	LINF_340017500	LmjF.34.1080	LinJ_34_1016	Amastin-like surface protein^P^	0.0066	− 1.0633
BSCL2mut	LINF_350036500	LmjF.34.1080	LinJ_35_3170	Hypothetical protein—conserved	0.0234	1.0013
BSCL2mut	LINF_070018400	NA	LinJ_07_1340	Amino acid transporter 19^P^	0.0012	1.0014
BSCL2mut	LINF_330015200	LmjF.33.0792^b^	LinJ_33_0864	Beta tubulin	0.0005	1.0104
BSCL2mut	LINF_330015100	LmjF.33.0792^b^	LinJ_33_0862	Beta tubulin	0.0001	1.0350
BSCL2mut	LINF_350009700	LmjF.34.1080	LinJ_35_0450	Generative cell specific 1 protein^P^	0.0018	1.0889
BSCL2mut	LINF_330015000	LmjF.33.0792^b^	LinJ_33_0860	beta tubulin	0.0001	1.0957
BSCL2mut	LINF_350014500	LmjF.35.0980	LinJ_35_1000	Aldose 1-epimerase^P^	0.0022	1.0992
BSCL2mut	LINF_230013000	LmjF.23.1665	LinJ_23_0700	PAP2 superfamily^P^	1.82e^−5^	1.3851

*NA* means not available.

^P^Means putative.

^a^In addition to the specified ID, it can be LmjF.34.1560, LmjF.34.1580, LmjF.34.1600, LmjF.34.1620, LmjF.34.1640, LmjF.34.1660, LmjF.34.1680, LmjF.34.1700, LmjF.34.1720, LmjF.34.1740, LmjF.34.1760, LmjF.34.1780, LmjF.34.1800, LmjF.34.1820, LmjF.34.1860, LmjF.34.1880, LmjF.34.1900, LmjF.34.1920, LmjF.34.1940.

^b^In addition to the specified ID, it can be LmjF.33.0794, LmjF.33.0796, LmjF.33.0798, LmjF.33.0800, LmjF.33.0802, LmjF.33.0804, LmjF.33.0806, LmjF.33.0808, LmjF.33.0810, LmjF.33.0812, LmjF.33.0814, LmjF.33.0816, LmjF.33.0818, LmjF.33.0819, LmjF.33.0820.

*L. infantum* expression in Infected BSCL2mut vs. WT showed 3 downregulated and 8 upregulated DEGs. These included downregulation of genes encoding for amastins, and upregulation of genes encoding an amino acid transporter 10 (*AAT19*), beta tubulin, generative cell specific-1 protein, aldose 1-epimerase, PAP2 superfamily (Table 1).

## Discussion

Individuals with CGL develop altered metabolism resulting in lipoatrophy, diabetes, liver pathology, and cardiovascular disease. They also have an increased risk of death due to infection^10^. *Leishmania* infection has already been shown to affect lipid metabolism, as evidenced by the buildup of lipid droplets in infected cells that are strongly linked to the parasite’s nuclei^22,23^. Here we present gene expression during *L. infantum* infection using an ex vivo model of macrophages from CGL type 1 and 2 individuals. There were changes in the transcriptional profile of both host and parasite genes. Macrophages herein studied have mutations in genes encoding the proteins AGPAT2 and Seipin, the first of which resembles a model of cells lacking the conversion of lysophosphatidic acid into phosphatidic acid, an important step in the synthesis of triacylglycerides, and the second by lipid droplets structural organization.

When macrophages are exposed to microbial stimuli, increased lipid synthesis is associated with enhanced phagocytosis^24^. Disruption in membrane lipids or lipid rafts may facilitate the entry of intracellular parasites and cause defective antigen presentation^10^. The category *membrane raft* was present in infected BSCL2mut. Antigen receptor-mediated signaling pathway, and downregulated *PRF1* and *TLR2* were present in both infected CGL types. In infected AGPAT2mut, *toll-like receptor signaling pathway* category was present and *IL1B* and *TLR8* were downregulated. Among the common DEGs of both infected CGL types, there was a sign of disruption in the immunological synapse, which was also an enriched category. Besides, genes involved in MHC-I, TCR-CD3 complex, apoptosis, IL-2, and LCK signaling pathways were downregulated. Secondary signaling pathways, such as *ICOS* and *CTLA4* might be facilitated by synapse formation and were downregulated in both CGL types as well^25^.

Changes in lipid metabolism are integrated with the signaling pathways that specify macrophage functions^24^. *L. infantum* influences the signaling pathways of the macrophages by interfering with the phosphorylation and activation status of various kinases from the JAK and MAPK families, affecting the transcription factors consequently (e.g. STAT1α, NF-κB). Protein tyrosine phosphatases (PTPs) have been demonstrated to be critical in the control of JAK/STAT pathways in regard to IFN-γ signaling^26^. Here, we found *PTPRF* upregulated, *JAK3*, *STAT1*, and *IFNGR2* downregulated, and also the category *tyrosine phosphorylation of STAT protein* in the AGPAT2mut MDMs. Besides, membrane structure and curvature depend on phospholipids, including PtdIns. Insulin resistance in CGL individuals might be related to AGPATs role in the biosynthesis of PtdIns, the substrate for PI3K (phosphatidylinositol-3-kinase), which acts on membrane integrity and provides substrates for insulin signaling^6^. Category *regulation of phosphatidylinositol 3-kinase signaling* is enriched on both CGL types. Irregular PtdIns production in CGL cells has an apparent role in the response of intracellular pathogen infection.

In both infected AGPAT2mut and BSCL2mut, the category of *clathrin-coated endocytic vesicle* was found. A patient with CGL type 4 failed to respond to a lipid nanoparticle encapsulated mRNA, but succeeded with Adenovirus-based vaccine (with entry mediated by clathrin-coated pits)^27^. Clathrin-mediated endocytosis may be a viable alternative way for the CGL cells to compensate for their lipid-related membrane trafficking impairment. Furthermore, dysregulation of phospholipid metabolism during endocytosis is associated with neuronal disorders. The enriched category *regulation of neuron death* for both uninfected and infected samples is possibly present in macrophages due to the similarity of the process of endocytosis and autophagy, that is essential in neurons for the proteins/organelles removal when they are no longer are useful. This is a plausible relationship between lipid metabolism and clinically observed cognitive impairment for both CGL types^28^. In uninfected BSCL2mut, there were categories of external side of plasma membrane, membrane invagination, and phagocytosis, also altered mental-disorder-related genes such as *TPSB2*, *GNAO1*, *HAPLN3*, and *DHCR7*.

There is an association between BSCL2mut and inflammation^29^, even without any infectious process involved. Immune-like genes were DEGs in both uninfected samples of AGPAT2mut (*P2RY6, CXCL9, HLA-F, AOAH*, all downregulated) and BSCL2mut (*GDF15* and *P2RY2* upregulated; *PTAFR*, *HLA-F*, *NOD1, NLRP1, CCL13, TNFSF10*, and *CXCL9* downregulated). Insulin resistance and diabetes mellitus 2 (DM2) are both central concerns in the clinic of CGL. A prevalence of 68.2% of DM2 is observed in CGL individuals in the state of Rio Grande do Norte, Brazil^5^. Here, we found genes related to insulin resistance and DM2 (*PTPRF* and *CTLA4* altered in BSCL2mut; *IGFBP4* altered in both CGL types). Adipocytes lacking caveolin are more fragile and show increased inflammation and collagen deposition^30^. In infected AGPAT2mut cells, *CAVIN1* was downregulated, and the *collagen-containing extracellular matrix* was a category of enrichment analysis. Inflammation and fibrosis are candidate biological processes that can explain the metabolic consequences of lipodystrophy. *COL4A1* gene was upregulated in AGPAT2mut, which encodes type IV collagen alpha protein, an integral component of basement membranes. This indicates a relationship between collagen metabolism and lipodystrophies. Therefore, it was possible to capture biochemical signals manifested systemically by investigating MDM as a tool, with DEGs related to the CGL clinic and phenotype identified.

Intriguingly, *CAVIN1* was downregulated in *L. infantum* infected AGPAT2mut. Mutations in the *CAVIN1* gene cause CGL type 4^6,28^. In addition to the established CGL subtypes, phenotypes that are closely related to it have been reported: mutation in genes encoding the proto-oncogene c-FOS protein (*FOS*)^3,28^. *FOS* was downregulated in AGPAT2mut and BSCL2mut, with and without *L. infantum* infection. Although CGL is caused by different mutations, there was a sign of combined CGL-related genes which might be involved in their increased risk of death because of infection^10^. Additionally, a RNA-seq of PBMCs with 7 CGL type 2 individuals showed one upregulated DEG in common with our CGL type 2 (BSCL2mut) MDMs: *CEBPE*. The CCAAT Enhancer Binding Protein Epsilon (*CEBPE*) is a transcriptional activator required in myeloid differentiation^31^.

Some clinical insights can be considered in the CGL Type 1 vs. CGL Type 2 comparison. *ADAM22* (upregulated in uninfected AGPAT2mut when compared to uninfected BSCL2mut) is implicated in cell–cell and cell–matrix interactions, and neurogenesis, and is highly expressed in the brain. *PTPRU* (downregulated in infected AGPAT2mut vs. infected BSCL2mut) is part of the protein tyrosine phosphatase (PTP) family, a family of signaling molecules. This PTP plays a role in cell–cell recognition and adhesion. This PTP is related to early neural development^32^. *VLDLR* (downregulated in both infected and uninfected AGPAT2mut vs. BSCL2mut) encodes a lipoprotein receptor, a member of the LDLR family, and plays main roles in VLDL-triglyceride metabolism and the reelin signaling pathway, which plays a crucial role in regulating neuronal migration and positioning during brain development^33^. *PRRC2A* gene (downregulated in infected AGPAT2mut when compared to BSCL2mut) has been demonstrated to control neural development^34^ and has microsatellite repeats associated with the age-at-onset of insulin-dependent diabetes mellitus^35^, important to CGL clinics.

Amastins are a gene family that codes surface proteins expressed in the *Leishmania* amastigote stage. Amastin seems to be directly involved in the interaction of intracellular parasites and host cell membranes. The absence of δ-amastins resulted in impaired growth of *L. braziliensis*^36^. We found δ amastin genes (see Supplementary Fig. S4) less expressed in *L. infantum* when infecting both CGL groups. *Leishmania* amastigotes form particularly tight junctions with the parasitophorous vacuole membrane (PVM) that might permit two-way transport of lipids between the host cell and amastigotes. These junctions contain parasite amastin proteins, as well as the Mø scavenger receptor, CD36, suggestive of a role in transporting fatty acids or other metabolites across the PVM^37^. Besides, lower amastin expression might impact the efficiency of promastigote-to-amastigote conversion, affecting the interaction of *Leishmania* with macrophages and the overall differentiation outcome. Iron metabolism and reactive oxygen species are involved in this differentiation, accompanied by an increase of amastigote-specific markers, such as amastin mRNAs^38–40^.

A *PAP2* superfamily gene was upregulated in the BSCL2mut MDMs, and is localized in the plasma membrane. It has been implicated in the conversion of external bioactive lipids, cellular signaling, vesicular trafficking, endocytosis, and stress response. It was demonstrated in *Leishmania guyanensis* that deletion of a PAP2-like gene leads to a disturbance of the cell cycle and affects the ratio of critical intracellular lipids^41^. Also localized in the membrane, there was amino acid transporter 19 (*AAT19*) as an upregulated gene in BSLC2mut. Different AATs have been characterized as related to distinct roles beyond the uptake of amino acids as nutrients, such as osmotic control, differentiation, and infection^42^. Besides, the vital virulence factor of leishmanolysin in *Leishmania* pathogenesis has been long documented^43,44^, being part of the gp63 multigene family. A mechanism by which *Leishmania* can subvert macrophages’ regulatory pathways to alter NF-kappaB activity was demonstrated during macrophages in vitro infection causing NF-kappaB p65 RelA cleavage. This cleavage is dependent on the *Leishmania* gp63^45^. *GP63* was upregulated in *L. infantum* infecting AGPAT2mut cells and also the category of *I-kappaB kinase/NF-kappaB signaling* was present in the AGPAT2mut MDMs.

Our study has some limitations. MDM AGPAT2mut was less prone to *Leishmania* infection since the relative number of MDM infected was low when compared to both WT and BSCL2mut (see Supplementary Fig. S2), without statistical difference among the number of infected MDM. As a result, the findings in this study may be due solely to differences in transcript abundance rather than changes in the number of cells examined. The difference in host response to infection is more likely to be caused by the metabolic derangement of the lipodystrophy, instead of cell-specific expression and/or isoforms of AGPAT2. Importantly, our data were from an in vitro infection by *L. infantum* during 72 h to perform RNA-seq. This time is appropriate for immunological synapse, but may not be feasible for a specific one. In the metatranscriptome analysis, the *Leishmania* complex was expected. The presence of primates could be due to sequencing technical errors, not detected in the quality assessment step, or to sequence differences between the reads from our samples and the human genome reference^46^. In vitro polarization has matter that the macrophage population is not necessarily representative of macrophages found in vivo, and a few in vivo settings reflect the constraints of in vitro polarization^47^. This study had a low sample size and lack of sample pairing, due to the rarity of CGL. Further functional analysis is required.

At the molecular level, lipodystrophy cells infected with an intracellular parasite are impaired in cellular communication, signaling, and membrane dynamics. *Leishmania* has evolved mechanisms to exploit host cell lipids for its own benefit. Lipid metabolism pathways in lipodystrophy cells influence the intracellular environment. Some of *L. infantum* DEGs found here, are localized in the membrane: amastins, PAP2, AAT19, and GP63, and play important roles in *Leishmania*’s ability to infect and persist within host cells, as well as in modulating the host's immune response.

In conclusion, genes involved in membrane trafficking, signaling and direct membrane-to-membrane interaction were modulated during the infectious process of CGL cells. Expression of membrane-associated genes was observed in phagocytosed *L. infantum*. The exact pathways in which the impaired phospholipids (due to mutations in *AGPAT2* and *BSCL2*) lead to impaired response to infection have yet to be determined, but here we propose candidates as: Endocytosis (e.g. clathrin-mediated endocytosis); collagen metabolism; immune synapse (e.g. antigen presentation, IFN-γ signaling, JAK/STAT); NF-kappaB signaling; and irregular PtdIns biosynthesis. It was possible to capture biochemical signals that are manifested systemically by using MDMs. Differentially expressed genes here without any infectious process are directly related to the clinical findings observed in individuals with lipodystrophy. In CGL cells, there is a sign of combined lipodystrophy-related genes with a pattern of downregulation of *CAVIN1* and *FOS*.

## Materials and methods

### Participants

Type 1, type 2 CGL, and Wild Type individuals were recruited between January 2015 and August 2016. All participants were previously genotyped for mutations shown to cause CGL, as follows: (1) Individuals diagnosed with CGL type 1, named *AGPAT2mut*, and defined as individuals homozygous for rs138994150 (c.646A<T, p.Lys216*), n = 3; (2) Individuals diagnosed with CGL type 2, named *BSCL2mut*, and defined as individuals homozygous for rs786205071 (c.517dupA, p.Thr173Asnfs*), n = 3; and (3) Wild Type individuals, defined as a healthy individual carrying two copies of the functional genes, with no prior history of visceral leishmaniasis, named WT, n = 4. CGL individuals were not on metreleptin treatment at the time they participated in this study, they were started a year afterward with support from the Brazilian Health Minister. Peripheral blood cells from each group were studied with or without in vitro* L. infantum* infection. An overview of the methodology can be found as Supplementary Fig. S1.

### Sample collection and in vitro assays

*Leishmania infantum* were isolated from bone marrow aspirate of visceral leishmaniasis subject from Natal, Brazil. *Leishmania* was typed by isoenzyme at a World Health Organization reference Center (Fundacao Oswaldo Cruz, Rio de Janeiro, RJ). After isolation, *L. infantum* were cryopreserved. In every experimental analysis, one vial of cryopreserved parasites was cultured in Schneider’s insect medium (Gibco, USA) containing with 1.5 μM l-glutamine (Sigma, USA) and 20% bovine fetal serum (Sigma, USA) at 25OC. Stationary promastigotes were harvested after three days of culture and used to infect monocytes-derived macrophages. In all experiments, stationary *L. infantum* to infection was P2–P5 passage.

Heparinized peripheral blood was collected from all studied individuals. Peripheral blood mononuclear cells (PBMC) were obtained after Ficoll Hypaque gradient. After overnight resting at 37 °C in 5% CO2, adherent monocytes were separated from PBMC. During 6 days at 37 °C in 5% CO2, adherent monocytes were maintained in RPMI 1640 supplemented with 1.5 μM l-glutamine, 1 mM Hepes, 10% heat-inactivated AB human serum and 5 ng/mL of M-CSF (Gibco, USA), to transform into monocyte-derived macrophages.

Monocyte-derived macrophages from CGL individuals and controls were infected with stationary *L. infantum* promastigote at a 5 parasites/cell ratio, then incubated for 2 h, and subsequently were washed with warm RPMI 1640 to remove free parasites. After an additional 72 h, infected or control MDM were suspended in 500 µL of Trizol and stored at − 80 °C for sequence analysis.

*L. infantum* infection was assessed in the participants by serology and qPCR. We determined the presence of anti-*leishmania* antibodies in the sera by ELISA method as previously described^48^. Additionally, *L. infantum* circulating DNA was measured by q-PCR as described elsewhere^49^ with modifications, *L. infantum* genomic gene MAG 1 was used as the parasite target sequence (FW-5ʹ-AGAGCGTGCCTTGGATTGTG-3ʹ; RV-5ʹ-CGCTGCGTTGATTGCGTTG-3ʹ and probe 5ʹ-FAM-TGCGCACTGCACTGTCG-3ʹ), while human PPIA (peptidyl-prolyl Isomerase A) sequence was used as target to certify integrity of host DNA (FW-5ʹ-CAAGACTGAGATGCACAAGTG-3ʹ, RV-5ʹGTGGCGGATTTGATCATTTGG3ʹ and probe 5ʹ-Cy5 AATTCACGCAGAAGGAACCAGACAGT-3ʹ) in a duplex reaction at concentration of 100 nM each primer and 5 nM each probe. The thermal cycling used was an initial denaturation at 95 °C for 2 min, followed by 40 repetitions at 95 °C for 15 s for denaturation, and at 60 °C for 60 s for annealing/extension.

### Total RNA extraction, quality control and sequencing

RNA stored in Trizol was extracted using the standard Trizol protocol followed by a further clean up step using membrane (RNeasy Micro Kit, Qiagen, USA). RNA was quantified using a NanoDrop spectrometer (NanoDrop Technologies), and quality of the RNA was determined by using Agilent Bioanalyzer (RNA Nano Chip, Agilent), and Qubit 2.0/3.0 fluorometer. RNA samples isolated from MDM were sequenced on an Illumina HiSeq 2000 platform generating readings of 75 bp in length in paired-end, for each sample three experimental replicates. Sequence analyses were performed at the University of Iowa DNA core facility.

### Sequence data analyses

Reads from the sequenced libraries were mapped to the human genome (GRCh38) obtained from ENSEMBL^50^ and to the *L. infantum* genome (GCA_900500625.1 and GTF 46) obtained from Ensembl Protists. Alignments were done using the HISAT2 aligner version 2.0.5^51^ with standard parameters. The mapping step generated SAM files that were compressed in BAM using Samtools version 1.3.1^52^. After compression, reading counts assigned to the gene regions of the reference genome, for each library, were accessed using the FeatureCounts program version 1.5.2^53^ with annotation file GFF3. Subsequent steps were done using R version 4.2^54^. Unexpressed and weakly expressed genes, defined as having at least 10 reads per line, were removed before further analysis, resulting in a final count table of 17,526 genes for human and 8716 for *L. infantum*.

### Analysis of differentially expressed genes

Differential gene expression was assessed with the DESeq2 package version 1.36.0^55^ from Bioconductor. Differentially expressed genes (DEGs) between paired conditions were identified as those with adjusted P value (BH) < 0.05 and absolute value of log2 fold change (Log2FC) > 1. Enriched pathways were identified using Gene ontology (GO)^56^, defined as those with a q value < 0.05 (Biological Process Level 3), or using KEGG pathways^57^. When needed REVIGO^58^ was used to filter non-redundant categories. DEGs Z-scaled log2 [CPM] (Counts per million mapped reads) values were plotted.

### Metatranscriptome analyses

For quality control of the samples, metatranscriptomic analysis was performed for all the groups of this study, including the infected and uninfected samples. At first, to list the mapped and unmapped reads to the human genome, Samtools^59^ was used. The unmapped reads to the human genome (the result of HISAT2 analysis described in the previous section) were aligned to a binary non-redundant (nr) protein database using DIAMOND^60^. Taxonomic identity was annotated in MEGAN6 version 6.8.18^61^. The resulting DIAMOND files were visualized and compared in MEGAN6.

### Ethical considerations

This study adhered to the norms and guidelines proposed by resolution 466/12 of the Brazilian National Ethical Committee, which regulates research involving human participants. The protocol was reviewed and approved by the Federal University of Rio Grande do Norte’s ethical committee (CAAE: 14070213.3.0000.5537). Overall approval was also obtained from the University of Iowa Institutional Review Board (protocol 200712745). Participants were informed about the purpose and steps of this study, and they or their legal guardians signed the informed consent.

### Supplementary Information

Supplementary Information.

Supplementary Table S1.

Supplementary Table S2.

Supplementary Table S3.

Supplementary Table S4.

## Data Availability

The data discussed in this publication have been deposited in NCBI’s Gene Expression Omnibus^62^ and are accessible through GEO Series accession number GSE210555 (https://www.ncbi.nlm.nih.gov/geo/query/acc.cgi?acc=GSE210555).
