# Supplementary material for: Impaired signaling pathways on Berardinelli–Seip congenital lipodystrophy macrophages during Leishmania infantum infection
Source: Sci Rep. 2024 May 16;14:11236. doi: 10.1038/s41598-024-61663-6 (PMC11099049; doi:10.1038/s41598-024-61663-6)
Supplement: Supplementary file 1 — Supplementary Information. [file 41598_2024_61663_MOESM1_ESM.pdf]

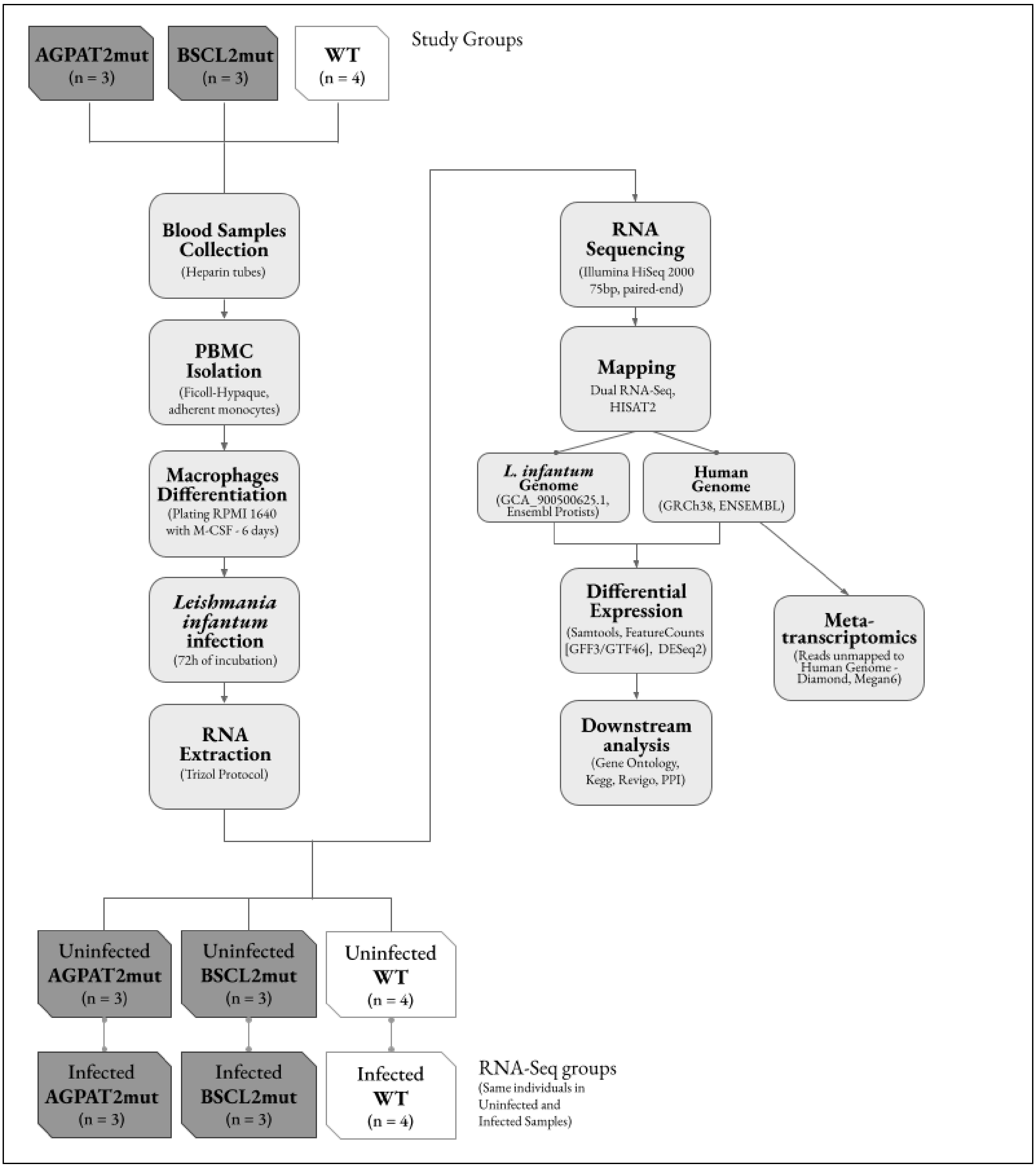

Supplementary Figure S1 – Methodology overview. AGPAT2 and BSCL2mut are type 1 and type 2 are Berardinelli-Seip syndrome (CGL) individuals, respectively. WT is the group for Wide Type individuals. *In vitro* and *In silico* workflow are summarized. *Leishmania infantum* infected and uninfected samples were included in the RNA-sequencing.

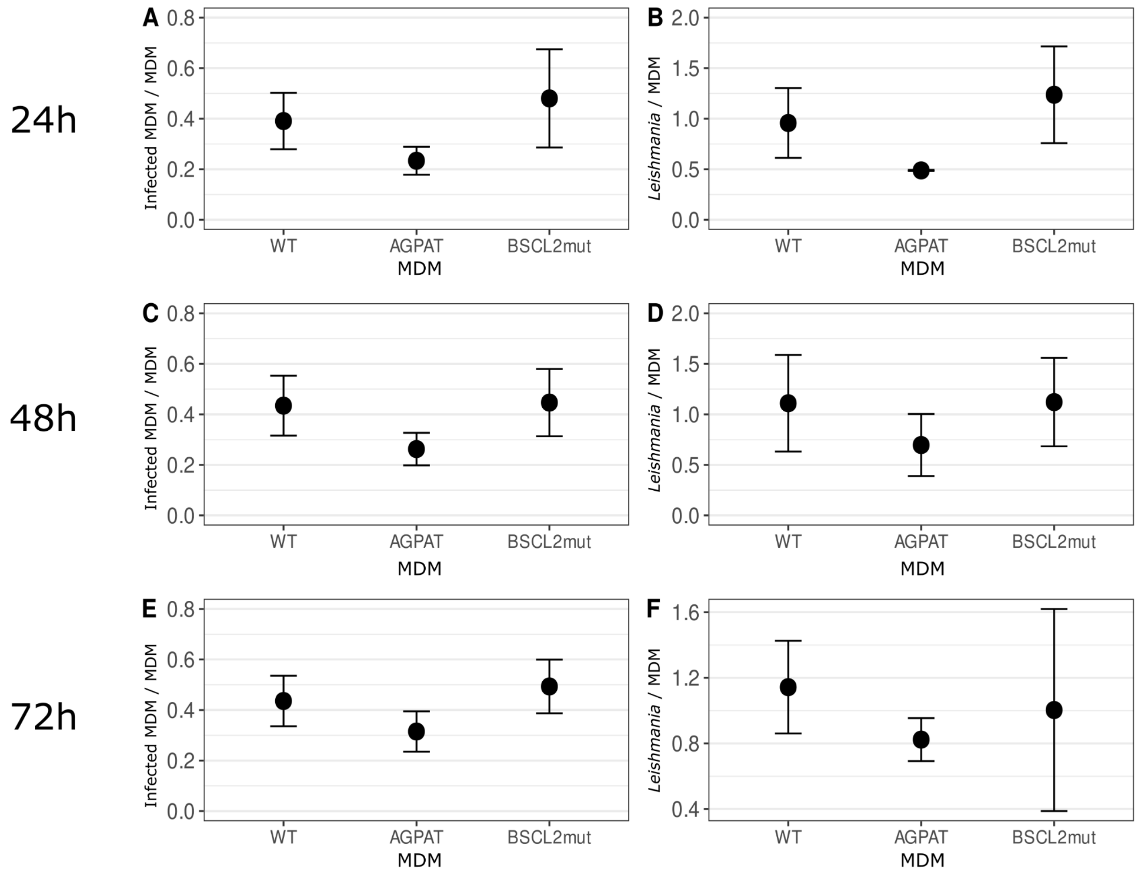

Supplementary Figure S2 - Relative numbers of intracellular parasites 24h, 48h, and 72h after MDM incubation with *Leishmania* promastigotes. The average of counted MDM per sample was 211.8 (SD=9.3). A, C, and E) Infected MDM relative to the number of MDM according to each MDM type. B, D, and F) Number of *Leishmania* amastigotes per MDM. *Different at $p < 0.05$ by ANOVA test.

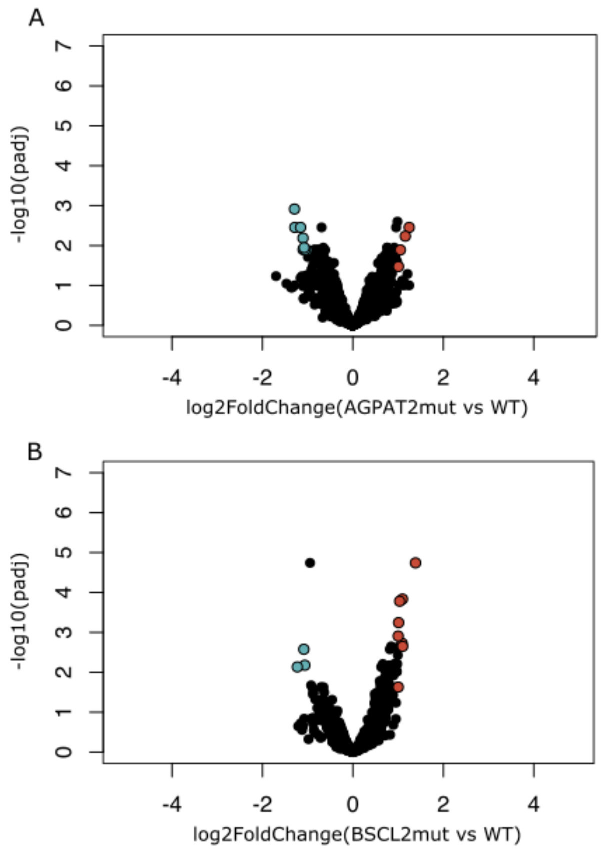

Supplementary Figure S3 - *L. infantum* gene expression. Each dot represents one gene in the volcano plot. DEGs (defined as Log2FoldChange > 1 and padj < 0.05) are represented by blue (downregulated) and red (upregulated) dots. A) Genes expressed by *L. infantum* in the comparison AGPAT2mut vs WT, with 8550 genes and 12 DEGs represented. B) Genes expressed by *L. infantum* in the comparison BSCL2mut vs. WT, with 8540 genes and 11 DEGs represented.

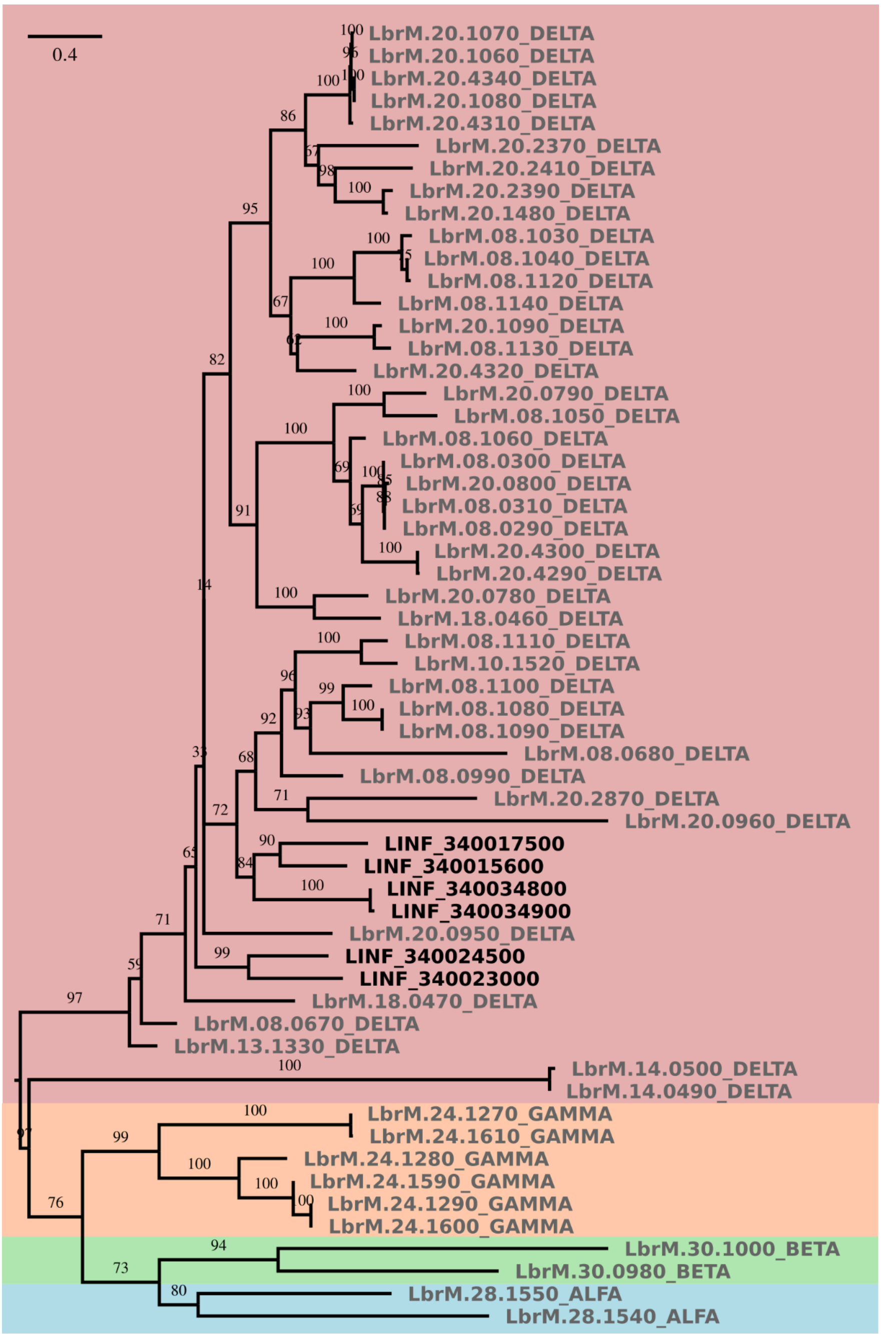

Supplementary Figure S4. Phylogenetic tree of amino acid sequences of 52 amastins from *L. braziliensis*, and 6 amastins differentially expressed in *L. infantum* in this study. *L. infantum* transcripts were obtained from TritrypDB and translated into amino acid using the software Aliview (the complete protein sequence was used). All sequences were aligned using MAFFT v7.409 (53) and the phylogenetic tree was estimated by the Maximum likelihood method by the IQ-TREE v2.2.0 (54) under Jones-Taylor-Thornton (JTT) model

(55). A consensus tree was built after 1000 bootstrap resampling. Branch lengths are drawn proportion to evolutionary change with bootstrap values shown on each node. Classification into alfa, beta, gamma, and delta amastin sub-families are shown on the colors and was based on Paiva (2015).

Supplementary Table S1 - Molecular and serological tests for *L. infantum*. Cutoff value for SLA (Soluble Leishmania antigen) was 0,375.

Supplementary Table S2 - Detailed information about Differentially Expressed Genes. DEGs found in the comparisons AGPAT2mut vs. WT (uninfected), BSCL2mut vs. WT (uninfected), AGPAT2mut vs. WT (infected with *L. infantum)*, BSCL2mut vs. WT (infected with *L. infantum*), AGPAT2mut vs. BSCL2mut (uninfected), and AGPAT2mut vs. BSCL2mut (infected with *L. infantum*).

Supplementary Table S3 - List of all enrichment analyses from the infected comparisons. Enriched pathways (defined as those with a q value < 0.05) from the comparisons of AGPAT2mut vs. WT (infected with *L. infantum*) and BSCL2mut vs. WT (infected with *L. infantum*), including cellular component, molecular process, and biological process of Gene Ontology.

Supplementary Table S4 - List of all enrichment analyses from the uninfected comparisons. Enriched pathways (defined as those with a q value < 0.05) from the comparisons of AGPAT2mut vs. WT and BSCL2mut vs. WT, including cellular component, molecular process, and biological process of Gene Ontology.
